# Quantitative proteomics by SWATH-MS reveals sophisticated metabolic reprogramming in hepatocellular carcinoma tissues

**DOI:** 10.1038/srep45913

**Published:** 2017-04-05

**Authors:** Yanyan Gao, Xinzheng Wang, Zhihong Sang, Zongcheng Li, Feng Liu, Jie Mao, Dan Yan, Yongqiang Zhao, Hongli Wang, Ping Li, Xiaomin Ying, Xuemin Zhang, Kun He, Hongxia Wang

**Affiliations:** 1 National Center of Biomedical Analysis, Beijing 100850, China; 2 Experiment Instrument Plant, Academy of Military Medical Sciences, Beijing 100850, China; 3 Translational Medicine Center of Stem Cells, 307-Ivy Translational Medicine Center, Laboratory of Oncology, Affiliated Hospital, Academy of Military Medical Sciences, Beijing 100071, China; 4 Beijing Shijitan Hospital, Capital Medical University, Beijing 100038, China; 5 Computational Omics Lab, Center of Computational Biology, Beijing Institute of Basic Medical Sciences, Beijing 100850, China

## Abstract

Hepatocellular carcinoma (HCC) is one of the most common cancers worldwide, and understanding its molecular pathogenesis is pivotal to managing this disease. Sequential window acquisition of all theoretical mass spectra (SWATH-MS) is an optimal proteomic strategy to seek crucial proteins involved in HCC development and progression. In this study, a quantitative proteomic study of tumour and adjacent non-tumour liver tissues was performed using a SWATH-MS strategy. In total, 4,216 proteins were reliably quantified, and 338 were differentially expressed, with 191 proteins up-regulated and 147 down-regulated in HCC tissues compared with adjacent non-tumourous tissues. Functional analysis revealed distinct pathway enrichment of up- and down-regulated proteins. The most significantly down-regulated proteins were involved in metabolic pathways. Notably, our study revealed sophisticated metabolic reprogramming in HCC, including alteration of the pentose phosphate pathway; serine, glycine and sarcosine biosynthesis/metabolism; glycolysis; gluconeogenesis; fatty acid biosynthesis; and fatty acid β-oxidation. Twenty-seven metabolic enzymes, including PCK2, PDH and G6PD, were significantly changed in this study. To our knowledge, this study presents the most complete view of tissue-specific metabolic reprogramming in HCC, identifying hundreds of differentially expressed proteins, which together form a rich resource for novel drug targets or diagnostic biomarker discovery.

Liver cancer is one of the most common malignant cancers in the world, with more than 850,000 new cases worldwide annually^1^. This neoplasm is currently the second leading cause of cancer-related death globally, and the incidence is increasing^2^. Among all primary liver cancers, hepatocellular carcinoma (HCC) is the most common neoplasm, accounting for approximately 90% of all cases^1^,^3^,^4^,^5^,^6^,^7^,^8^. Hepatitis B virus (HBV) infection, hepatitis C virus (HCV) infection, alcohol abuse and intake of aflatoxin B1 are the main factors contributing to HCC^1^,^3^,^4^,^5^,^6^,^7^. In China, HCC has been ranked as the second most frequent fatal cancer since the 1990s^9^, and the majority of HCCs in China are caused by HBV infection^10^,^11^.

Currently, surgical resection and liver transplantation are considered the best treatment options for early-stage HCC and are curative therapies for approximately 30% to 40% of early-stage patients^3^,^12^. Due to the asymptomatic features of HCC at early stages, patients are often diagnosed at very advanced stages. Thus, there is an urgent need to find key carcinogenesis-associated molecules for HCC diagnosis and treatment.

Mass spectrometry (MS)-based proteomic analysis of human clinical tissues is a powerful tool to investigate cancer biomarkers and therapeutic targets^13^. Numerous clinical studies of HCC have been reported over the past decade using various quantitative techniques^14^,^15^,^16^,^17^,^18^,^19^,^20^, including SILAC (stable isotope labelling by amino acids in cell culture), iTRAQ (isobaric tags for relative and absolute quantification) and CDIT (culture-derived isotope tags) labelling techniques as well as label-free proteomics approaches based on quantification by ion intensity or spectral counting. Label-free approaches are relatively cheap compared to labelling approaches; when labelling reagents are not required, high-throughput and sensitive analyses in a mass spectrometer are possible. Quantitative studies of HCC using spectral counting and ion intensities have also been reported^19^,^20^. SWATH-MS (sequential window acquisition of all theoretical mass spectra) is an emerging label-free quantification approach that combines a highly specific data independent acquisition (DIA) method with a novel targeted data extraction strategy to mine the resulting fragment ion data sets. SWATH-MS has been widely used to compare protein expression and modify alterations^21^,^22^,^23^,^24^. To our knowledge, no SWATH-MS approach has been used to study HCC proteomics until now.

In this study, we compared the protein expression of tumourous (HCC) and adjacent non-tumourous (non-HCC) tissues from 14 HBV-associated HCC patients using a SWATH-MS technique to identify new HCC biomarkers and potential therapeutic target candidates. In total, 338 differential proteins were quantified, and most down-regulated proteins were involved in metabolism. Sophisticated reprogramming of cell metabolic pathways was revealed. These observations are essential to elucidate the mechanisms underlying the occurrence and progression of HCC and contribute to the discovery of new candidates for early HCC diagnosis.

## Results

### Differentially expressed proteins quantified by SWATH-MS analysis in HCC tissues

**Figure 1:**
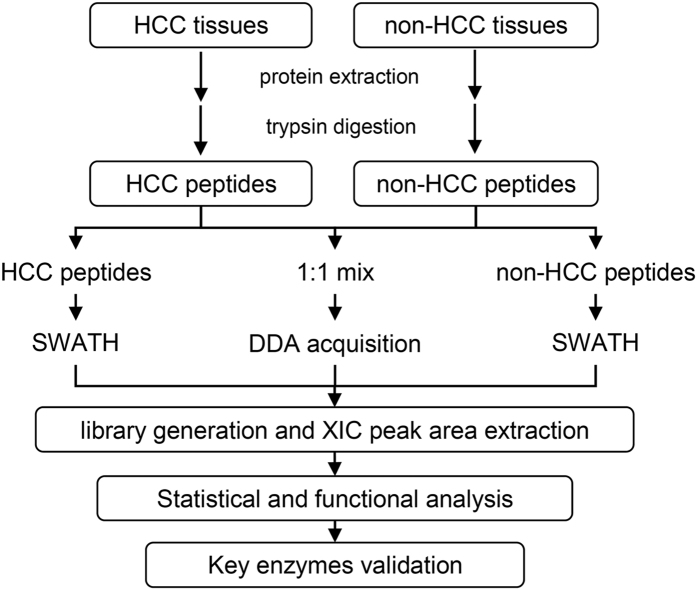
Quantitative proteomic workflow of human HCC and adjacent non-tumourous liver tissues analysed using a SWATH-MS approach.

The experimental scheme of the present study is shown in Fig. 1. HCC and non-HCC liver tissue samples were compared by SWATH-MS to identify differentially expressed proteins that can be used as biomarkers for HCC diagnosis or in HCC development and progression. To avoid individual differences and detect true HCC-related proteins, samples were analysed by equal pooling of two or three tissues from both groups to determine a quantitative expression ratio between HCC and non-HCC liver tissue groups based on total ion intensity normalization. Five biological replicates were analysed, and 14 pairs of tissue samples were used in total (Supplementary Table S1).

The targeted identification of peptides in SWATH-MS datasets requires *a priori* generation of a spectral library that includes essential coordinates for each targeted peptide, such as precursor ion masses, fragment ion masses, fragment ion intensities and retention times^21^. For each biological replicate, a spectral library was generated with a traditional data-dependent acquisition (DDA) mass spectrometry technique as described in the Methods section. Five libraries were obtained in total. On average, the spectral libraries contained approximately 2,491 distinct protein groups, and 26,586 peptides were identified with greater than 99% confidence and passed the global false discovery rate (FDR) from fit analysis using a critical FDR of 1% (Supplementary Table S2, Supplementary Figure S1). Taken together, these findings indicated that the experimentally generated spectral libraries contained only high-confidence proteins.

**Figure 2:**
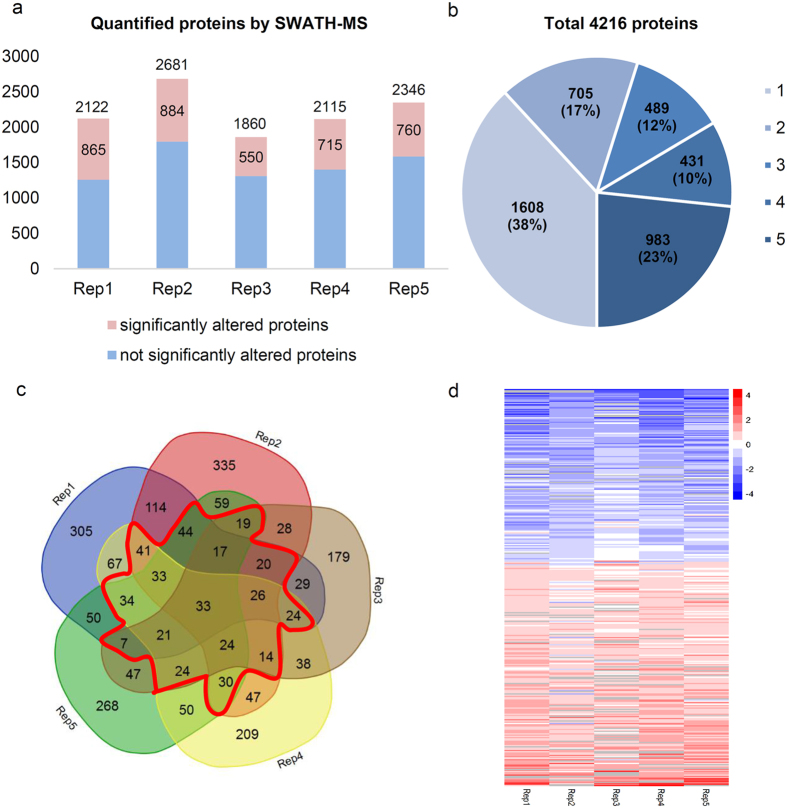
Quantitative proteomic analysis between HCC and non-HCC tissues. (**a**) Numbers of proteins quantified by SWATH-MS in each biological replicate. Larger and smaller numbers in each column indicate total quantified and significantly changed proteins (*p* < 0.05, fold change (FC) ≥1.5 or FC ≤ 1/1.5) in each biological replicate. Rep1 through Rep5 are abbreviations of biological replicates 1 through 5. (**b**) Protein distributions were quantified one to five times. Numbers in parentheses indicate the percentage of total proteins. Legend numbers 1–5 on the right side show the repeat times of the quantified proteins. (**c**) Venn diagram depicting overlap of significantly regulated proteins (*p* < 0.05, FC ≥1.5 or FC ≤ 1/1.5) in five biological replicates. Red lines show proteins significantly regulated in at least 3 of 5 biological replicates. (**d**) Heatmap of 338 significantly regulated proteins, including 191 up-regulated and 147 down-regulated proteins. The colour bar on the right side represents the expression level of the proteins, corresponding to log2-ratios of FC (HCC/non-HCC). Red indicates up-regulation, and blue indicates down-regulation.

Following generation of the spectral library, the identification and quantification of HCC and non-HCC proteins were performed using a SWATH-MS approach as described by Gill *et al*.^21^, with modifications. Proteins were digested by trypsin, and the peptides were separated using a gradient of 120 min on a reverse-phase nanoLC instrument. SWATH data from six injections for each biological replicate were submitted in unison to PeakView software (Version 1.2, AB Sciex) for targeted data extraction, which resulted in the quantitative export of 2,122, 2,681, 1,860, 2,115 and 2,346 unique proteins for the analysis of five biological replicates (Fig. 2a). In total, 4,216 proteins were quantified in at least one biological replicate, and 1,903 proteins were quantified in at least three biological replicates, which accounted for 45% of all quantified proteins (Fig. 2b and Supplementary Table S3).

Relative protein quantification was analysed using MarkerView (Version 1.2.1, AB Sciex) and R (Version 3.3.1, the R foundation), as described in the Methods section. Normality distribution of each technical replicates was performed after log2 transformation for the peak intensities of all the MS measurements prior to further analysis and the histograms were shown in Supplementary Figure S2. In total, four criteria were used to filter out differential proteins. First, the Shapiro-Wilk test was used to test normality for each protein within one biological replicate, and only proteins that met normality were used for further analysis. Second, Welch’s t-test and Benjamini-Hochberg multiple test correction were performed, and an adjusted *p*_value < 0.05 and fold change (FC) ≥ 1.5 or FC ≤ 1/1.5 was considered statistically significant. With these two criteria, 865, 884, 550, 715 and 760 differentially expressed proteins were obtained for the five biological replicates (Fig. 2a). Third, up- or down- regulated proteins were detected in at least three biological replicates are shown in the Venn diagram (Fig. 2c). Fourth, the average ratio of up- or down-regulated proteins had to meet the 1.5-FC cutoff requirements. With the above mentioned four criteria, 191 up-regulated and 147 down-regulated proteins were obtained in total and are shown in the heatmap (Fig. 2d). Supplementary Table S4 and Table S5 present the differentially expressed proteins for the adjusted *p*_value < 0.05 and FC ≥1.5 or FC ≤ 1/1.5 in five biological replicates and the final analysis results.

To evaluate our SWATH-MS data, comprehensive proteomics-based quantitative expression data obtained from five previously published studies of HCC and adjacent/normal tissues were used to perform side-by-side comparisons^15^,^17^,^18^,^20^,^25^. The differentially expressed proteins quantified in these five studies were 151, 573, 71, 267 and 648. The comparison revealed that 49 (42), 155 (150), 28 (26), 68 (65) and 70 (62) differentially expressed proteins detected in our data were present in the five previous studies; the trends of the major proteins were consistent with published data (numbers in brackets represent proteins with the same change trend) (Supplementary Table S6). In total, 214 (199) proteins in our dataset were detected in previous studies, which accounted for 63.31% (58.88%) of the 338 differential proteins. Among the 214 proteins, 99 proteins were detected in ≥3 studies, including our data (Supplementary Table S6). Among these 99 frequently identified common proteins, 41 proteins were up-regulated, and 58 were down-regulated. Among the 338 differentially expressed proteins, nine were validated by western blotting in previous studies, and five were validated by immunohistochemical (IHC) methods. Seven of the nine western blotting-validated proteins (OTC, PEBP1, CPS1, BHMT, CLIC1, PPA1 and APEX1) and all five IHC-tested proteins (OTC, BHMT, CLIC1, PPA1 and APEX1) were detected in ≥3 data sets. All five proteins (OTC, BHMT, CLIC1, PPA1 and APEX1) were tested using both methods as HCC potential biomarkers. Our SWATH data were consistent with the western blotting- and IHC-validated results (Supplementary Table S6).

To decipher whether the differentially expressed proteins were detectable in plasma, we searched for these proteins in the Human Plasma Proteome database (HPPD)^26^. Approximately 85.50% of the differentially expressed proteins (289/338) appeared in this database, suggesting that they had relatively strong potential to be secreted into the blood (Supplementary Table S7). Among these 289 proteins, the numbers of up- and down-regulated proteins were 169 and 120, respectively. Furthermore, 94 proteins were detected in ≥3 studies and were present in HPPD; among these, 38 proteins were up-regulated (Supplementary Table S7). These proteins were potential biomarker candidates for HCC; some may be tested using multiple reaction monitoring (MRM), as described by Hou *et al*.^27^. The above results show the reliability of our SWATH-MS proteomic results and indicate that these differentially expressed proteins may be useful to delineate HCC properties and screen HCC biomarker candidates.

### GO and KEGG pathway enrichment analysis

To investigate the function of these differentially expressed proteins, GO and KEGG pathway analyses of up-regulated and down-regulated proteins were performed separately by DAVID (Version 6.8, LHRI & DAVID Bioinformatics)^28^,^29^. This method easily determined the characteristics of up- or down-regulated proteins. Liver proteins downloaded from the human proteome map (http://humanproteomemap.org) were used as the background dataset for enrichment analysis^30^. For up-regulated proteins, 34 significant enrichments were identified using GO analysis (*p* < 0.05, *p*_values were corrected using the Benjamini-Hochberg procedure). These were classified into three GO categories, including biological processes (BP, 5), molecular functions (MF, 6) and cellular components (CC, 23) (Table 1). In BP, those items significantly participated in cell-cell adhesion (*p* = 7.12 × 10^−5^), mRNA splicing via the spliceosome (*p* = 4.60 × 10^−3^), SRP-dependent cotranslational protein targeting to membrane (*p* = 6.97 × 10^−3^) and translation initiation (*p* = 9.07 × 10^−3^). The most significant terms for MF and CC were poly(A) RNA binding (*p* = 5.60 × 10^−20^) and extracellular exosome (*p* = 1.30 × 10^−15^), respectively. For the down-regulated proteins, 66 significant enrichments were obtained, including 29 BP, 28 MF and 9 CC (Table 1). The five most significant terms of BP were related to metabolic processes, including oxidation-reduction processes (*p* = 4.93 × 10^−21^), xenobiotic metabolic processes (*p* = 1.03 × 10^−8^), metabolic processes (*p* = 1.95 × 10^−8^), drug metabolic processes (*p* = 3.32 × 10^−8^) and epoxygenase P450 pathway (*p* = 7.15 × 10^−7^). For MF and CC, the most significant terms were oxidoreductase activity (*p* = 1.03 × 10^−16^) and mitochondrial matrix (*p* = 1.69 × 10^−18^), respectively.

**Figure 3:**
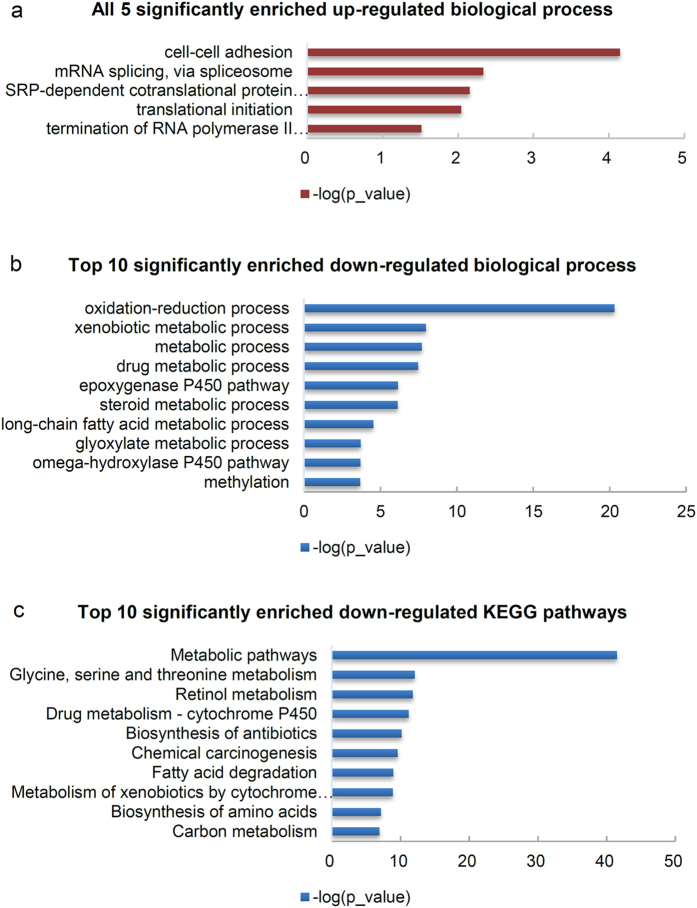
GO and KEGG pathway enrichment of 338 significantly regulated proteins according to DAVID functional annotation. (**a**) All 5 and significantly enriched biological processes of up-regulated proteins quantified using the SWATH-MS approach. (**b,c**) Top 10 significantly enriched biological processes and KEGG pathways of down-regulated proteins quantified using the SWATH-MS approach.

To locate the key pathways implicated in HCC development and progression, KEGG pathway enrichment analysis was performed for 338 differentially expressed proteins. As for GO analysis, up- and down-regulated proteins were analysed separately, and the background of the enrichment used liver proteins from the human proteome map^30^. Pathway analysis showed that the up-regulated proteins were significantly enriched in term of spliceosome (*p* = 4.24 × 10^−2^). For down-regulated proteins, 37 terms were enriched, and the most significant terms were metabolic pathways (*p* = 3.36 × 10^−42^), glycine, serine and threonine metabolism (*p* = 9.03 × 10^−13^), retinol metabolism (*p* = 1.65 × 10^−12^), drug metabolism - cytochrome P450 (*p* = 6.72 × 10^−12^) and biosynthesis of antibiotics (*p* = 7.05 × 10^−11^). The all five significant GO terms of the BP for up-regulated proteins and the top 10 most significant GO terms of the BP and KEGG pathways for down-regulated proteins are shown in Fig. 3(a–c). Those of MF and CC are in Supplementary Figure S3(a–d). All enriched terms are shown in Supplementary Table S8.

### Western blot validation for nine selected proteins in clinical HCC tissues

**Figure 4:**
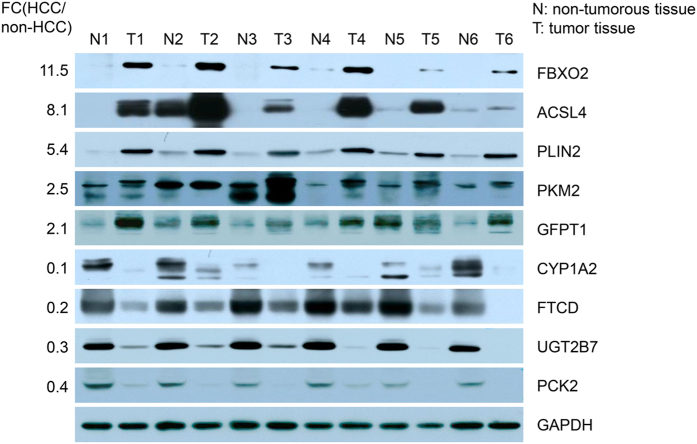
Validation of nine selected proteins in clinical HCC tissues by western blotting. The abundance of FBXO2, ACSL4, PLIN2, PKM2, GFPT1, CYP1A2, FTCD, UGT2B7 and PCK2 proteins in HCC and adjacent non-HCC liver tissues were analysed by western blotting using six pairs of samples. The GAPDH protein was used as an internal reference.

Nine candidate proteins were selected for validation by western blot analysis using six sample pairs of HCC and non-HCC liver tissues that differed from those used in the proteomics studies (Supplementary Table S1 patient ID 15–20). The candidate proteins were selected based on either dramatic fold change or involvement in key metabolic pathways. Five candidate proteins were up-regulated, namely FBXO2, ACSL4, PLIN2, PKM2 and GFPT1. The four down-regulated proteins were CYP1A2, FTCD, UGT2B7 and PCK2. Here, the analysis showed differential expression of all the candidates in HCC tissues compared with non-HCC tissues. FBOX2, ACSL4 and PLIN2 showed strong expression in all six tumour samples but weak or no expression in non-tumour tissues. PKM2 and GFPT1 showed generally high expression levels in five HCC tissues compared with the control group. For all down-regulated proteins, low expression was detected in HCC tissues compared with non-HCC tissues (Fig. 4). The representative extracted ion chromatogram (XIC) comparisons in these nine proteins are shown in Supplementary Figure S4. Overall, the western blot analysis results for all nine proteins were consistent with the proteomics data, which indicated that our proteomics data were highly reliable and that some proteins are worthy of further investigation.

## Discussion

The purpose of this study was to characterize proteomic changes in HCC tissues, provide potential protein candidates for biomarker discovery and suggest molecular mechanisms of HCC development and progression. Although much proteomic research has been performed, the biological mechanisms of HCC development and progression are still unclear.

Metabolic reprogramming is a hallmark of cancer^31^. Cancer cells can increase the amount of glucose and glutamine to satisfy energy needs and macromolecular synthesis demands. Therefore, understanding the metabolism of tumours remains an intense study topic with important therapeutic potential^32^.

**Figure 5:**
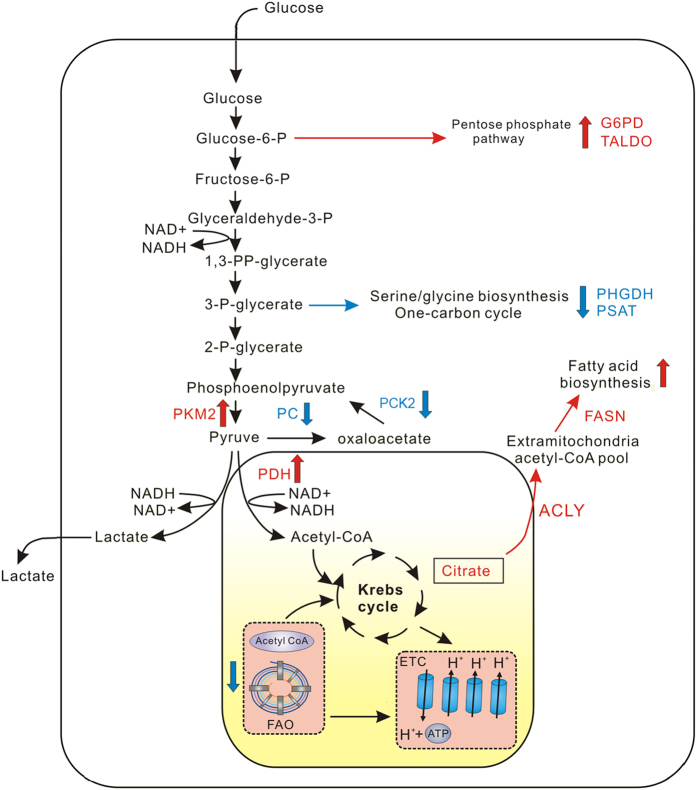
Sophisticated metabolic reprogramming in HCC. Our proteomic data revealed the up-regulation of glycolysis and pentose phosphate pathways and fatty acid biosynthesis and the down-regulation of gluconeogenesis; serine, glycine and sarcosine metabolism; and fatty acid β-oxidation. The red letters and arrows indicate up-regulated proteins and pathways, respectively, and the blue letters and arrows indicate down-regulated proteins and pathways.

Using the newly developed SWATH-MS technique, we quantified more than 4,000 proteins, and 338 proteins were differentially expressed in HCC. Sophisticated metabolic reprogramming was revealed as depicted in Fig. 5, including the following major aspects.

First, the oxidative pentose phosphate pathway (PPP) was up-regulated in HCC. PPP is the first branch pathway of glycolysis. In PPP, glucose-6-phosphate becomes partially oxidized to generate NADPH and ribose-5-phosphate. PPP is frequently elevated in tumourigenesis. In our study, two key enzymes—the rate-limiting enzyme glucose-6-phophate dehydrogenase (G6PD) and transaldolase (TALDO)—were over-expressed in HCC. The over-expression of G6PD and TALDO was detected in previous HCC references^18^,^25^,^33^,^34^.

Second, the serine, glycine and sarcosine biosynthesis/metabolism pathways were significantly down-regulated in HCC. Serine biosynthesis is a key metabolic pathway for cell proliferation, contributing carbon to many anabolic products, such as protein, glutathione, nucleotide and phospholipid biosynthesis^35^,^36^. Phosphoglycerate dehydrogenase (PHGDH) and phosphoserine aminotransferase (PSAT) are two key enzymes of serine biosynthesis^37^,^38^. In our study, both PHGDH and PSAT were down-regulated in HCC tissues. The two proteins were quantified in the HCC proteomics study mentioned above, with the same trends. Overall, our data indicate that the serine biosynthesis pathway is down-regulated in HCC.

In line with PHGDH and PSAT, key enzyme of serine/glycine metabolism, serine hydroxymethyl-transferase 1 (SHMT1) in the cytosol was down-regulated. SHMT catalyses the formation of glycine from serine, thereby generating 5,10-methylene-tetrahydrofolate (5,10-MTHF)^39^. Glycine-N-methyl transferase (GNMT), the enzyme that generates sarcosine from glycine, showed dramatically low expression. In addition to GNMT, sarcosine levels were regulated by sarcosine dehydrogenase (SARDH), the enzyme that converts sarcosine back to glycine and dimethylglycine dehydrogenase (DMGDH), which generates sarcosine from dimethylglycine^40^. Both SARDH and DMGDH were down-regulated in HCC tissues. In addition, betaine-homocysteine S-methyltransferase 1 (BHMT1) converts betaine and homocysteine into dimethylglycine and methionine. In our study, BHMT1 was also down-regulated.

**Figure 6:**
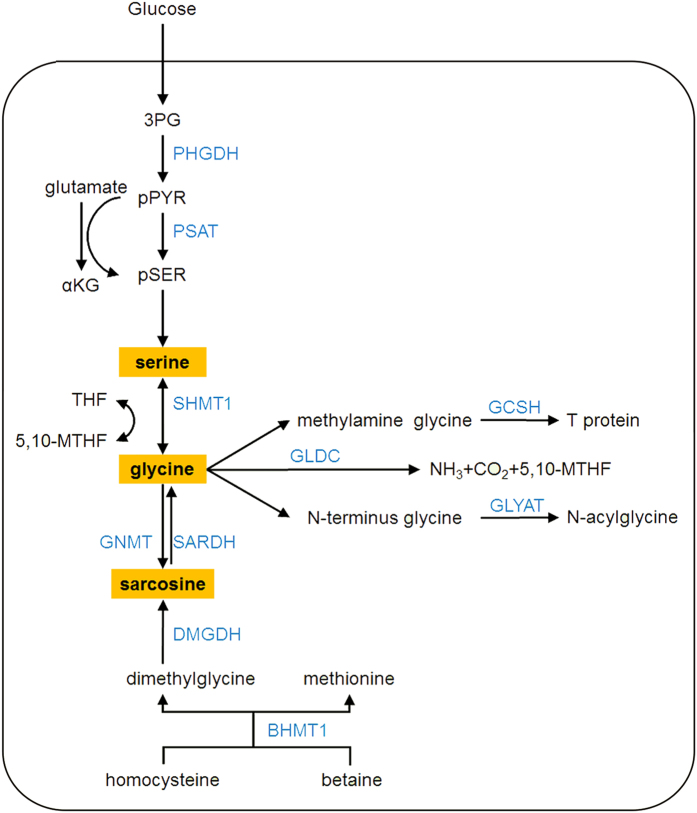
Down-regulation of serine, glycine and sarcosine biosynthesis/metabolism pathways in HCC. Ten enzymes involved in serine, glycine and sarcosine metabolic pathway were differentially expressed in our study and all were down-regulated. The down-regulated proteins are shown in blue.

In addition to generating sarcosine, the glycine cleavage system is a catabolic mechanism for glycine. Three key glycine catabolism enzymes—glycine decarboxylase (GLDC), glycine cleavage system H protein (GCSH) and glycine N-acyltransferase (GLYAT)—were also decreased in HCC tissues. GLDC is a key component of the highly conserved glycine cleavage system in amino acid metabolism, which catalyses the breakdown of glycine to form CO_2_, NH_3_ and 5,10-MTHF to fuel one-carbon metabolism^41^. GLDC and GCSH are two members of the glycine cleavage system, and their down-regulation indicated the significantly low expression of the system. In total, 10 serine, glycine and sarcosine metabolic enzymes were down-regulated in our study. The schematic pathway and changes in these enzymes are shown in Fig. 6.

PHGDH is the first enzyme in serine biosynthesis from a glycolic intermediate, is frequently amplified in breast cancer and is required for the growth of PHGDH-amplified cells *in vitro*^37^. The over-expression of GLDC in the tumour-initiating cells (TIC) of non-small cell lung cancer (NSCLC) was also reported to induce dramatic changes in glycolysis and glycine metabolism^42^. Sarcosine was identified as a differentially expressed metabolite that was highly elevated during prostate cancer progression to metastasis and is a potentially important metabolic intermediate of cancer cell invasion and aggression^40^. These studies showed that the up-regulation of serine/glycine metabolism may contribute to the pathogenesis of several human cancers and may provide novel targets for improving anticancer therapies.

Overall, our observation was the opposite of earlier results, which may be due to different cancer types. GLDC, together with GCSH and eight other enzymes related to glycine metabolism, mainly catabolism, were down-regulated in HCC. This indicated that glycine catabolism plays different roles in HCC other than NSCLC, and the function of glycine metabolism in HCC is worthy of further investigation. The down-regulation of the glycine catabolism pathway agreed well with the increased glycine in HCC serum compared with normal control samples in a recent metabolomics study^43^.

Third, fatty acid synthesis (FAS) was up-regulated in HCC. Fatty acid *de novo* synthesis is required for membrane synthesis and therefore for cell growth and proliferation. It has been revealed that fatty acid synthesis requires several key enzymes, including pyruvate dehydrogenase (PDH) complex, citrate synthase (CS), ATP-citrate synthase (ACLY), acetyl-CoA carboxylase (ACC) and fatty acid synthase (FASN)^44^. Three of these, ACLY, ACC and FASN, were frequently up-regulated in transformed cells^45^. In our study, one subunit of PDH, CS, ACLY and FASN were all over-expressed in HCC. The over-expression of these four enzymes, especially the dramatic increase in ACLY and FASN, indicated the up-regulation of fatty acid biosynthesis in HCC. An increased capacity for producing lipids *de novo* facilitates the formation of lipid bilayers but also enables the cell to adapt to oxidative stress^46^. We speculate that similar mechanisms exist in HCC for fatty acid synthesis as in other cancers.

Moreover, fatty acid oxidation (FAO, also known as β-oxidation) was down-regulated in HCC. In most cases, the growth and survival of cancer cells is limited by levels of cytosolic NADPH. The production of FAO-derived cytosolic NADPH by cancer cells is critical to counteract oxidative stress^47^. In our study, 6 enzymes of FAO were quantified. Levels of all of these enzymes were decreased, including ACADS and ACADSB (which catalyse acyl-CoA dehydrogenase activity); ECHS1, ECHD2 and ECHD3 (which catalyse enoyl-CoA hydratase activity); and ACAA2 (which catalyse acetyl-CoA C-acyltransferase activity). The schematic diagrams of FAO and all altered enzymes are shown in Supplementary Figure S5. Evidence indicates that some tumours, including prostate tumours, leukaemia and large B-cell lymphoma, utilize FAO as their main energy supply for proliferation and survival^48^. The down-regulation of FAO in HCC indicated that FAO plays different roles in HCC, unlike other tumours.

In addition to the above mentioned pathways, glycolysis and gluconeogenesis are key metabolic pathways in human cells. Conversion of glucose to lactate is a major pathway of glucose metabolism in cancer cells even in the presence of O_2_; this is known as the Warburg effect or aerobic glycolysis^49^. Pyruvate kinase (PK) catalyses the final rate-limiting reaction in glycolysis by transferring high-energy phosphate from phosphoenolpyruvate (PEP) to ADP to produce ATP and pyruvate^50^. PKM2 is the dominant M isoform in most adult tissues and is the major PK in proliferating and cancer cells^50^. Elevated PKM2 expression has been demonstrated in various human tumours, including lung, breast, prostate, blood, cervix, kidney, bladder and colon, when compared to matched normal tissues^51^. PKM2 promotes tumourigenesis by regulating the Warburg effect, as reported in a previous study^50^. In our proteomic data, PKM2 was over-expressed in HCC tissue, and the expression of PKM2 was also validated by western blotting. Thus, our results indicate that the change in PKM2 in HCC is consistent with the Warburg effect.

As shown in Fig. 5, pyruvate can be transformed into two different products, acetyl-CoA and oxaloacetate, by PDH and pyruvate carboxylase (PC). PDHA1, a pyruvate dehydrogenase E1 component subunit alpha, was over-expressed, while PC was down-regulated in our proteomic data set. The down-regulation of PC in HCC has been reported^20^. Thus, our data indicated that the increase in pyruvate was directed to acetyl-CoA for the TCA cycle, which may further be used for fatty acid synthesis.

Phosphoenolpyruvate carboxykinase (PCK) is the rate-limiting enzyme of gluconeogenesis in the liver and kidney. There are two isoforms of PCK: cytosolic PCK (PCK1) and a mitochondrial isoform of PCK (PCK2). PCK1, but not PCK2, was reported to be over-expressed in colorectal cancer and promotes tumour growth by increasing glucose and glutamine metabolism^52^. PCK2 expression was reported to be elevated in non-small-cell lung carcinoma (NSCLC) and is regulated by glucose and required for *in vivo* tumour growth^53^. The down-regulation of PCK2 in HCC has been documented in a previous study^17^. In our proteomic data set, both PCK1 and PCK2 were lowly expressed in HCC. The down-regulation of PCK2 was validated using western blot analysis and was consistent with the proteomic data. Thus, the down-regulation of PCK was a specific characteristic of HCC, different from colorectal cancer and NSCLC in the literature. This observation is in agreement with the view that cancer cells display metabolic flexibility, and not all features are universal^53^. Taken together, these data demonstrate roles for PKM2, PC and PCK2 in HCC, which links metabolic flux and anabolic pathways to cancer cell proliferation.

In summary, we compared the proteomic profile of HCC tumour tissues (n = 14) with paired adjacent non-tumourous liver tissues (n = 14) using a SWATH-MS quantification strategy. To obtain reliable results, five biological replicates were performed, and strict filtration criteria were applied. In total, 338 differentially expressed proteins were obtained, and these proteins were involved in various key pathways. Compared with previous studies, more than 50% of proteins were quantified by others with the same change trend. Approximately 85% of the differentially expressed proteins showed relatively strong potential to be secreted into the blood. It is noteworthy that sophisticated metabolic reprogramming in HCC was revealed by proteomic data, including the up-regulation of glycolysis, the pentose phosphate pathway and fatty acid biosynthesis and the down-regulation of gluconeogenesis; serine, glycine and sarcosine metabolism; and fatty acid-oxidation. This reprogramming showed the distinct metabolic flexibility of HCC. In total, 27 key metabolic enzymes were quantified in our study, including PCK2, PDH and G6PD, which are important for cancer development and progression. In addition to metabolic reprogramming, spliceosome pathway was significantly up-regulated. The results indicate that the SWATH-MS strategy is effective for identifying crucial proteins involved in HCC development and progression. Differentially expressed proteins form a rich resource for diagnostic biomarkers or therapeutic drug target discovery. Furthermore, our findings into HCC-specific metabolic reprogramming may provide new insights into understanding HCC biology and direct optimal treatment.

## Materials and Methods

### Patients and Clinical Specimens

The following methods were carried out in accordance with approved guidelines. Access to human tissues complied with the guidelines of the Ethics Committee at Beijing 302 Hospital, and informed consent was obtained from all patients for collecting the specimens. All experimental protocols were approved by the National Center of Biomedical Analysis. HCC tissues and adjacent non-tumourous liver tissue counterparts used for this study were collected from 20 HBV-associated HCC patients who underwent hepatectomy at Beijing 302 Hospital between 2013 and 2014 (14 males and 6 females). The tumourous and non-tumourous liver tissues were determined by the experienced pathologist and pathological sections. None of these patients received anti-neoplastic therapy prior to surgery. All patients used for the quantitative proteomics study and western blotting verification had liver cirrhosis. Patients details are shown in Supplementary Table S1. Tissues were immediately snap-frozen in liquid nitrogen after surgical resection and stored at −80 °C until use.

### Reagents and Materials

Hepes, NaCl, Urea and thiourea were purchased from Sigma-Aldrich (St. Louis, MO, USA). Cocktail was purchased from Roche Diagnostics (Indianapolis, IN, USA). Trypsin were purchased from Promega (Madison, WI, USA). Protein Assay Dye Reagent Concentrate was purchased from Bio-Rad (Hercules, CA, USA). Dithiothreitol (DTT) was purchased from Amresco (Solon, OH, USA). Iodoacetamide (IAA) was purchased from Acros Organics (Morris Plains, NJ, USA). Acetonitrile (ACN, HPLC grade) and methanol were purchased from Fisher Scientific (Fair Lawn, NJ, USA). Formic acid (FA) was provided by Fluka (Milwaukee, WI, USA). SuperSignal West Pico was purchased from Thermo Scientific (Rockford, IL, USA). All water used in the experiments was purified using a Milli-Q system (Millipore, Billerica, MA, USA).

### Protein Extractions and Trypsin Digestion

For each extraction, ~0.2 g of tissue was ground into powder in liquid nitrogen with a pre-cooled mortar and pestle. Samples were homogenized on ice in 1 ml of lysis buffer (50 mM Hepes, 6 M urea, 2 M thiourea and 1 × protease inhibitor cocktail). The samples were lysed by sonication for 1 min on ice (pulse on 3 s, pulse off 10 s). After centrifugation at 14,000 × g for 30 min at 4 °C, the supernatant was collected, and the protein concentration was determined by the Bradford method^54^ (Bradford Protein Assay, Bio-Rad). Twenty μg proteins from each sample were digested with trypsin using filter-aided sample preparation (FASP) as previously described^55^,^56^. After digestion, the peptides were dried in a vacuum for MS analysis.

### Mass Spectrometry Analysis

MS analysis was performed using an AB Sciex 5600^+^ TripleTOF mass spectrometer (Concord, Ontario, Canada) interfaced to an Ekspert^TM^ NanoLC 425 system (Dublin, CA) as previously described^21^,^22^,^23^,^24^. For library construction, peptides were trapped on a NanoLC pre-column (Chromxp C18-LC-3 μm, size 0.35 × 0.5 mm, Eksigent), eluted onto an analytical column (C18-CL-120, size 0.075 × 150 mm, Eksigent) and separated by a 120-min gradient from 5 to 35% Buffer B (Buffer A: 2% ACN, 98% H_2_O, Buffer B: 98% ACN, 2% H_2_O, 0.1% FA) at a flow rate of 300 nL/min. Full-scan MS was performed in positive ion mode with a nano-ion spray voltage of 2.3 kv from 350 to 1500 (m/z), with up to 50 precursors selected for MS/MS (m/z 100–1500). The selection criteria for parent ions included an intensity greater than 150 counts/s, a charge state from +2 to +5, a mass tolerance of 50 mDa and dynamic exclusion for 15 s. Ions were fragmented in the collision cell using rolling collision energy.

In SWATH™ acquisition, the parameters were essentially the same as those described by Gillet *et al*.^21^. With the same chromatographic conditions used in the DDA run described above and a variable isolation window obtained using variable window package software (including 1 Da for the window overlap), a set of 55 overlapping windows was constructed, covering the precursor mass range of 400–1250 Da. The collision energy for each window was determined based on the appropriate collision energy for a 2+ ion centred in the window with a spread of 15 eV. The high-sensitivity mode was used, allowing accurate extraction of the fragment ion masses.

### Generating the Reference Spectral Library

For each bio-replicate analysis, three DDA injections were performed to increase protein coverage as described by Hou *et al*.^27^. All three mass spectrometry files were searched in unison using ProteinPilot software (Version 4.2, AB Sciex) with the Paragon algorithm as described by Haverland *et al*. with minor modifications^22^. Samples were input as unlabelled samples with the following parameters: iodoacetamide-cysteine alkylation, digestion with trypsin and no special factors. The search was conducted using a thorough identification effort and the human UniProt database (April 2013 release)^57^.

### SWATH-MS Data Analysis

Spectral alignment and targeted data extraction of DIA samples were performed with the SWATH Processing Micro App in Peakview (Version 1.2, AB Sciex) using the reference spectral library generated above as described by Haverland *et al*. with modifications^22^. There was a reference library for each bio-replicate comparison. Six DIA raw files in one comparison group were loaded in unison using an extraction window of 15 min and the following parameters: 5 peptides, 8 transitions and peptide confidence of >99%, including shared peptides and XIC width set at 50 ppm. After data processing, three distinct files were exported for further quantitation analysis. The processed mrkvw files containing protein information from PeakView were loaded into MarkerView (Version 1.2.1, AB Sciex) for normalization of protein intensity (peak area) for all runs using the built-in total ion intensity sum plug-in. Log2 transformation was performed prior to further statistical analysis. We plot the histogram to check the normality distribution of each technical replicate. Differential analysis was performed using R (Version 3.3.1, the R foundation). In each biological replicate, normality tests of protein expression were performed for HCC/non-HCC groups using the Shapiro-Wilk normality test. Proteins whose expression values met the normality in both groups were retained for Welch’s t-test and the Benjamini-Hochberg multiple test correction. Mean values of protein expression were used for calculation of fold change (FC). Proteins with adjusted *p* < 0.05 and FC ≥ 1.5 or FC ≤ 1/1.5 in at least three biological replicates and average FC ≥ 1.5 or FC ≤ 1/1.5 were regarded as differentially expressed proteins in this study.

### Functional Analysis

Protein IDs were converted to gene names using the UniProt Retrieve/ID mapping tool^57^. Heatmaps for expression of selected proteins in the five groups were created using the R (Version 3.3.1, the R foundation) heatmap package. The DAVID webserver (Version 6.8, LHRI & DAVID Bioinformatics) was employed for Gene Ontology enrichment analysis and KEGG pathway analysis^28^,^29^.

### Western Blotting Validation

Prior to western blotting, the protein concentration of the samples was determined by the Bradford assay method^54^. Equal amounts of 20 μg protein per sample were separated by SDS-PAGE on an 8–12% polyacrylamide gel. Proteins were subsequently transferred to PVDF membranes (Immun-Blot PVDF, Bio-Rad, Hercules, CA, USA), and membranes were blocked with 5% (w/v) skim milk in Tris-buffered saline with 0.1% Tween 20 (TBS-T) for 1 h at room temperature. The following antibodies were used: ACSL4 (1:1000), ADPR (1:1000), CYP1A2 (1:1000), FTCD (1:1000), FBXO2 (1:300) and UGT2B7 (1:100) from Proteintech (Rosemont, IL, USA); PKM2 (1:1000) and GFPT1 (1:1000) from Cell Signaling (Danvers, MA, USA); PCK2 (1:1000) from Abcam (Cambridge, MA, USA). These primary antibodies were diluted in 5% (w/v) skim milk in TBS-T and incubated with membranes overnight at 4 °C. After washing for five minutes in TBS-T three times, horseradish peroxidase-labelled secondary antibodies (Jackson ImmunoResearch, West Grove, PA, USA) were used for detection for 1 h at room temperature. Visualization of the immunoreactive proteins was accomplished using enhanced chemiluminescence (SuperSignal West Pico, Thermo, Rockford, IL, USA) followed by exposure to X-ray film (XBT, Carestream, Xiamen, Fujian, China).

## Additional Information

**Accession codes:** All the raw data and meta data were deposited in a public repository iprox database (www.iprox.org) with the accession IPX0000859000, which is based on the National Center for Protein Sciences, Beijing (the PHOENIX) Center).

**How to cite this article**: Gao, Y. *et al*. Quantitative proteomics by SWATH-MS reveals sophisticated metabolic reprogramming in hepatocellular carcinoma tissues. *Sci. Rep.* **7**, 45913; doi: 10.1038/srep45913 (2017).

**Publisher's note:** Springer Nature remains neutral with regard to jurisdictional claims in published maps and institutional affiliations.

## Supplementary Material

Supporting Information

Supplementary Dataset 1

Supplementary Dataset 2

Supplementary Dataset 3

Supplementary Dataset 4

Supplementary Dataset 5

Supplementary Dataset 6

Supplementary Dataset 7

Supplementary Dataset 8

## Figures and Tables

**Table 1:** GO and KEGG pathway analyses of up-regulated and down-regulated proteins by DAVID.

Enrichment terms	Up-regulated proteins enrichment (*p* < 0.05)	Down-regulated proteins enrichment (*p* < 0.05)
GO: BP	5	29
GO: MF	6	28
GO: CC	23	9
KEGG pathway	1	37
Total	35	103

BP, biological processes; CC, cellular components; MF, molecular functions.
