# Supplementary material for: Quantitative proteomics by SWATH-MS reveals sophisticated metabolic reprogramming in hepatocellular carcinoma tissues
Source: Sci Rep. 2017 Apr 5;7:45913. doi: 10.1038/srep45913 (PMC5381110; doi:10.1038/srep45913)
Supplement: Supporting Information [file srep45913-s1.pdf]

Supporting Information

Quantitative proteomics by SWATH-MS reveals sophisticated metabolic reprogramming in hepatocellular carcinoma tissues

Yanyan Gao[1,2], Xinzheng Wang[1], Zhihong Sang[1], Zongcheng Li[3], Feng Liu[1], Jie Mao[1], Dan Yan[4], Yongqiang Zhao[1], Hongli Wang[1], Ping Li[1], Xiaomin Ying[5], Xuemin Zhang[1], Kun He[1,*] & Hongxia Wang[1,*]

[1] National Center of Biomedical Analysis, Beijing 100850, China

[2]Experiment Instrument Plant, Academy of Military Medical Sciences, Beijing 100850, China

[3]Translational Medicine Center of Stem Cells, 307-Ivy Translational Medicine Center, Laboratory of Oncology, Affiliated Hospital, Academy of Military Medical Sciences, Beijing 100071, China.

[4] Beijing Shijitan Hospital, Capital Medical University, Beijing 100038, China

[5]Computational Omics Lab, Center of Computational Biology, Beijing Institute of Basic Medical Sciences, Beijing 100850, China

* **Correspondence and requests for materials should be addressed to K.H.** (Email: hk@proteomics.cn, Tel: 8610-66930306, Fax: 8610-68186281) **& H.W.** (Email: whx@proteomics.cn, Tel: 8610-66930342, Fax: 8610-68186281)

a **Rep1 FDR Analysis**

Protein Level FDR Analysis

Proteins Identified at Critical False Discovery Rates

	Number of Proteins Detected		
**Critical FDR**	**Local FDR**	*Global FDR*	**lobal FDR from Fit**
**1.0%**	*2386*	*2626*	**2623**
**5.0%**	**2476**	*2889*	*2906*
**10.0%**	**2518**		

** It is recommended you use numbers in bold and avoid using numbers in italics.*

b **Rep2 FDR Analysis**

Protein Level FDR Analysis

Proteins Identified at Critical False Discovery Rates

	Number of Proteins Detected		
**Critical FDR**	**Local FDR**	*Global FDR*	**lobal FDR from Fit**
**1.0%**	*2218*	*2481*	**2493**
**5.0%**	**2338**	*2730*	*2723*
**10.0%**	**2396**		

** It is recommended you use numbers in bold and avoid using numbers in italics.*

c **Rep3 FDR Analysis**

Protein Level FDR Analysis

Proteins Identified at Critical False Discovery Rates

	Number of Proteins Detected		
**Critical FDR**	**Local FDR**	*Global FDR*	**lobal FDR from Fit**
**1.0%**	*1763*	*1986*	**2021**
**5.0%**	**1885**	*2232*	*2229*
**10.0%**	**1942**	*2429*	*2431*

** It is recommended you use numbers in bold and avoid using numbers in italics.*

d **Rep4 FDR Analysis**

Protein Level FDR Analysis

Proteins Identified at Critical False Discovery Rates

	Number of Proteins Detected		
**Critical FDR**	**Local FDR**	*Global FDR*	**lobal FDR from Fit**
**1.0%**	*2238*	*2358*	**2355**
**5.0%**	**2240**	*2627*	*2636*
**10.0%**	**2245**	*2811*	*2852*

** It is recommended you use numbers in bold and avoid using numbers in italics.*

e **Rep5 FDR Analysis**

Protein Level FDR Analysis

Proteins Identified at Critical False Discovery Rates

	Number of Proteins Detected		
**Critical FDR**	**Local FDR**	*Global FDR*	**lobal FDR from Fit**
**1.0%**	*2231*	*2508*	**2493**
**5.0%**	**2348**	*2735*	*2733*
**10.0%**	**2399**		

** It is recommended you use numbers in bold and avoid using numbers in italics.*

Supplementary Figure S1. High quality spectral libraries were generated with the traditional DDA mass spectrometry technique for five biological replicates. Proteins filtered with 1% critical FDR (false discovery rate) were used for SWATH-MS data analysis. Rep1 to Rep5 are abbreviations of biological replicate 1 to 5.

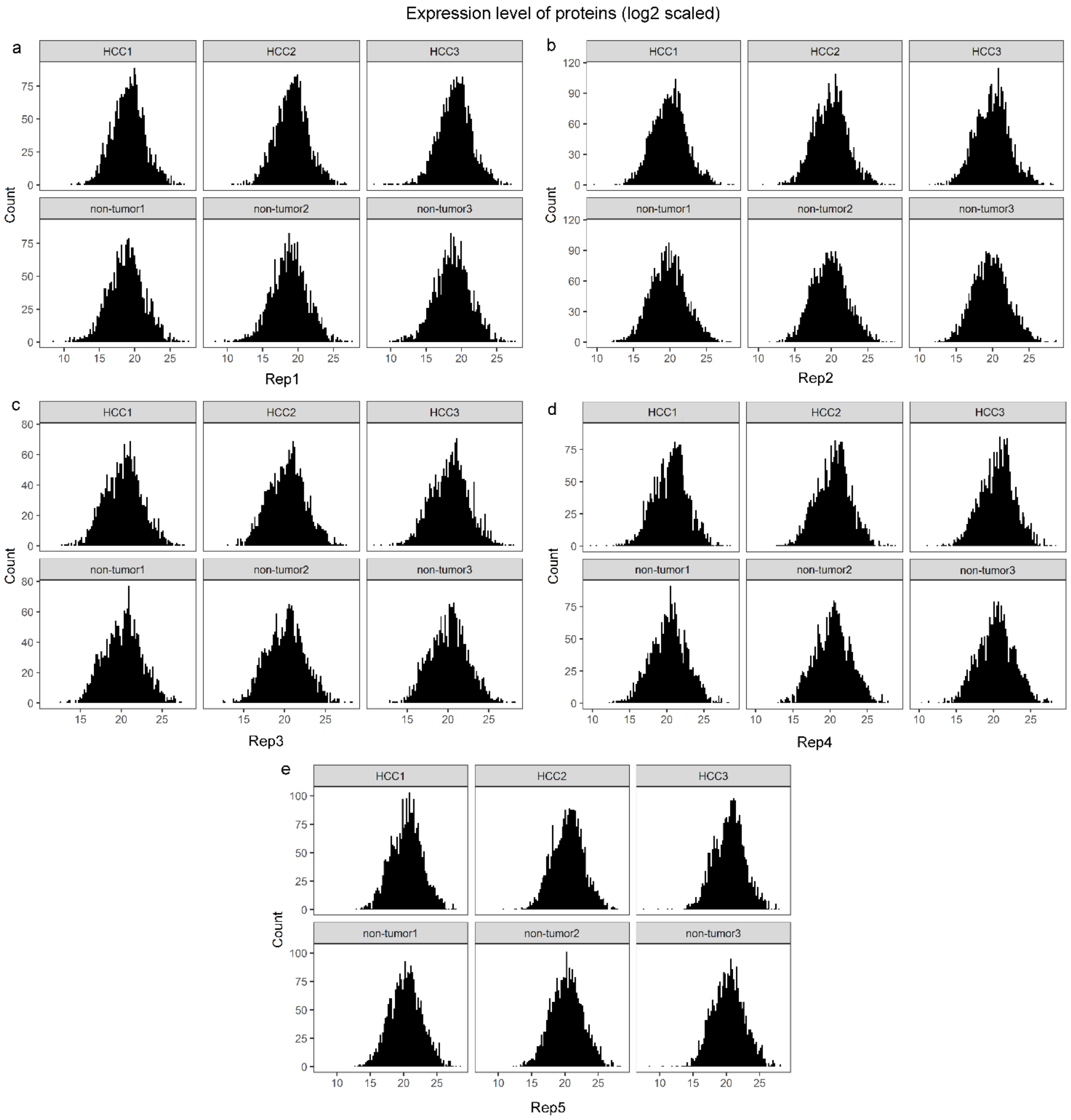

Supplementary Figure S2. Normality distribution of each technical replicates. The histograms was performed after log2 transformation for the peak intensities of all the mass spectrometry measurements. Rep1 to Rep5 are abbreviations of biological replicate 1 to 5. In each biological replicate, HCC1 to HCC3 are three technical replicates for HCC sample, and non-tumor1 to non-tumor3 are the three corresponding paired non-tumor technical replicates.

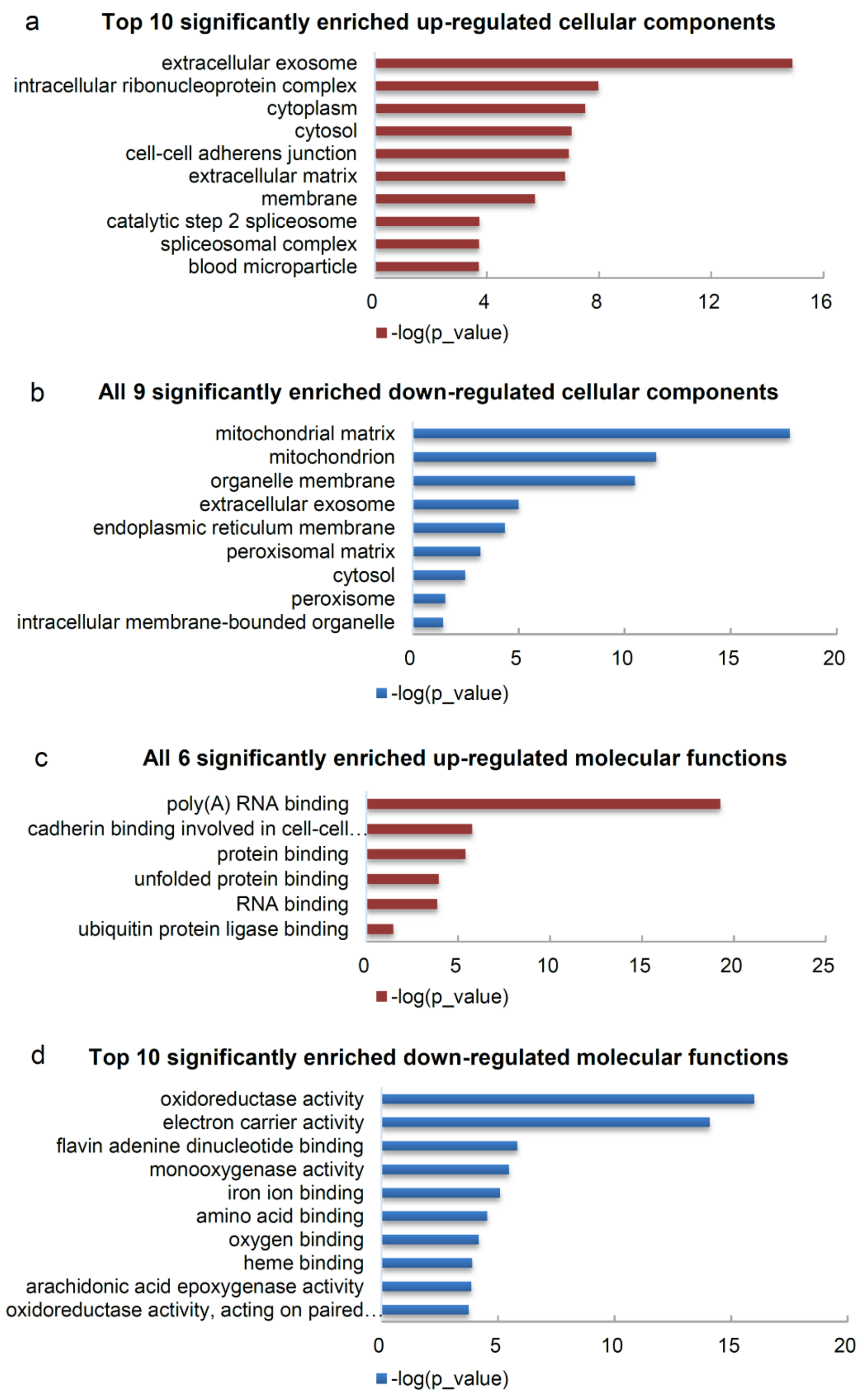

Supplemental Figure S3. GO enrichment of the 338 significantly regulated proteins according to DAVID functional annotation. (a, b), Top 10 and all 9 significantly enriched cellular components of up- and down-regulated proteins quantified using the SWATH-MS approach. **(c,d),** All 6 and top 10 significantly enriched molecular functions of up- and down-regulated proteins quantified using the SWATH-MS approach.

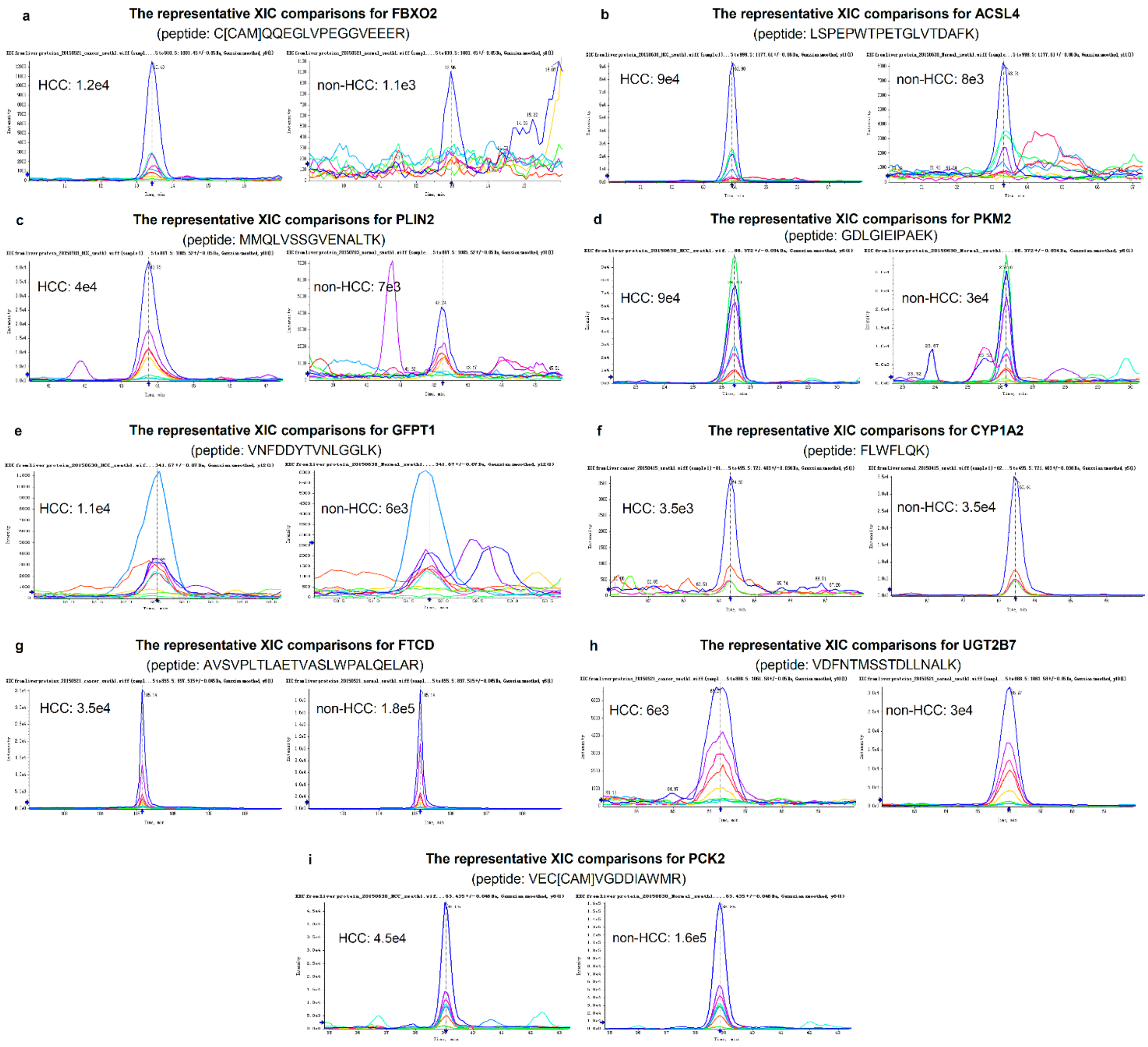

Supplemental Figure S4. The representative extracted ion chromatogram (XIC) comparisons of nine proteins selected for western blot validation (FBXO2, ACSL4, PLIN2, PKM2, GFPT1, CYP1A2, FTCD, UGT2B7 and PCK2). The fragment intensities for one peptide of each protein were shown. The first five proteins were over expressed in HCC and the last four proteins were low expressed in HCC.

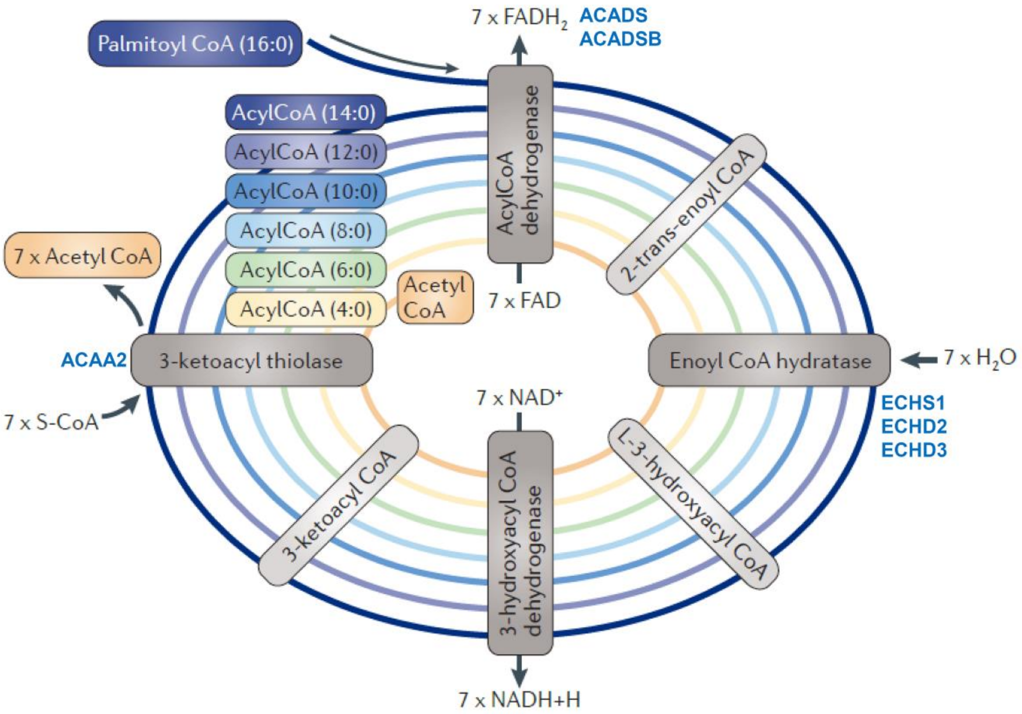

Supplemental Figure S5. The schematic diagram of fatty acid oxidation (FAO) and its down regulation in HCC. Acyl CoAs enter the FAO pathway in which they are dehydrogenated, hydrated and decarboxylated cyclically, which results in the progressive shortening of the fatty acid[1]. Six enzymes of FAO were down regulated in this study, including two acyl-CoA dehydrogenase (ACADS and ACADSB), three enoyl-CoA hydratase (ECHS1, ECHD2 and ECHD3) and one 3-ketoacyl-CoA thiolase (ACAA2).The abbreviations of down-regulated proteins were shown in blue. (Cited and modified from Carracedo, Cantley& Pandolfi[1] with permission.)

Reference

1. Carracedo, A., Cantley, L. C. & Pandolfi, P. P. Cancer metabolism: fatty acid oxidation in the limelight. *Nat Rev Cancer***13**, 227-232 (2013).
